# Oncolytic virus–mediated GPC3 delivery overcomes antigen heterogeneity in hepatocellular carcinoma and enhances GPC3-directed immunotherapy

**DOI:** 10.3389/fimmu.2026.1894178

**Published:** 2026-09-18

**Authors:** Shyambabu Chaurasiya, Sang-In Kim, Prakash Sah, Zhifang Zhang, Yoya Vashi, Hannah Wu, Jennifer Cillis, Anthony Park, Annie Yang, Yanghee Woo, Guangyang Xiong, Jingbao Liu, Hongbing Zhang, Pei Wang, Cheng Liu, Yuman Fong

**Affiliations:** 1 Depratment of Surgery, City of Hope National Medical Center, Duarte, CA, United States; 2 Department of Microbiology and Molecular Genetics, Oklahoma State University, Stillwater, OK, United States; 3 Eureka Therapeutics Inc, Emeryville, CA, United States

**Keywords:** bite, CF33, codrituzumab, glypican-3, HepG2, ECT204, ARTEMIS T cells

## Abstract

**Background:**

Hepatocellular carcinoma (HCC) remains a major cause of cancer-related mortality worldwide and is characterized by substantial molecular and immunologic heterogeneity that contributes to therapeutic resistance and poor clinical outcomes. Glypican-3 (GPC3) is an attractive immunotherapy target in HCC; however, its heterogeneous expression limits the effectiveness of GPC3-directed approaches. ECT204, a GPC3-directed ARTEMIS^®^ T-cell therapy, has shown improved safety compared with conventional CAR T cells but remains dependent on sufficient tumor-associated GPC3 expression. In this study, we investigated whether an oncolytic virus could be used to selectively deliver GPC3 to tumor cells to expand the applicability of GPC3-targeted immunotherapies.

**Methods:**

We engineered the oncolytic vaccinia virus CF33-GPC3 to express GPC3 and evaluated its ability to sensitize GPC3-negative tumor cells to ECT204 *in vitro* and *in vivo* using GPC3-knockout HepG2 xenograft models. We also assessed the compatibility with other GPC3-targeted immunotherapies, including a bispecific T-cell engager (BiTE) and the monoclonal antibody codrituzumab.

**Results:**

CF33-GPC3 efficiently induced surface expression of GPC3 in previously GPC3-negative tumor cells, resulting in activation of GPC3-directed ECT204 T cells and subsequent tumor cell killing. *In vivo*, combination therapy with CF33-GPC3 and ECT204, administered either intratumorally or intravenously, achieved significantly greater tumor control than either monotherapy alone. Moreover, OV-mediated GPC3 expression sensitized tumor cells to killing by GPC3-specific BiTE-redirected T cells and enhanced codrituzumab-mediated NK cell cytotoxicity.

**Conclusions:**

These findings demonstrate that OV-mediated GPC3 delivery can overcome antigen heterogeneity in HCC and broaden the applicability of GPC3-directed immunotherapies.

## Introduction

Cancer remains a major global health challenge and is currently the second leading cause of death worldwide (1). Solid tumors account for nearly 90% of all malignancies, representing the dominant contributor to cancer-related morbidity and mortality. Among these, liver cancer is of particular concern, ranking as the sixth most commonly diagnosed cancer globally and exhibiting a steadily rising incidence (1). In the United States, liver cancer incidence has tripled over the past four decades (American Cancer Society: *Cancer Facts and Figures*, 2025). Hepatocellular carcinoma (HCC) constitutes the predominant form of primary liver cancer and is frequently diagnosed at advanced stages, when curative options are limited and prognosis remains poor (2, 3).

Despite advances in surgical resection, ablation, locoregional therapies, and systemic treatments—including multi-kinase inhibitors and immune checkpoint inhibitors (ICIs)—clinical outcomes for most patients remain suboptimal. Key barriers include late-stage diagnosis, the paucity of tumor-specific therapeutic targets, and a profoundly immunosuppressive tumor microenvironment that collectively drive therapeutic resistance and disease progression (4–6). These limitations underscore an urgent need for more effective and durable immunotherapeutic strategies.

Over the past decade, cancer treatment has been transformed by immune-based therapies. Immune checkpoint blockade, chimeric antigen receptor (CAR) T cells, bispecific T-cell engagers (BiTEs), monoclonal antibodies, and antibody–drug conjugates (ADCs) have demonstrated the ability to harness immune effector mechanisms with unprecedented specificity (6–10). While these approaches have achieved remarkable success in hematologic malignancies, their translation to solid tumors has been comparatively limited. A major contributing factor is the availability of lineage-restricted antigens in blood cancers, such as CD19 in B-cell malignancies, which is uniformly expressed and has enabled the clinical success and regulatory approval of multiple CD19-directed CAR-T cell therapies and the BiTE blinatumomab (11–13). In contrast, solid tumors rarely express uniformly distributed, tumor-specific antigens, posing a fundamental challenge to the development of effective and safe immune-redirecting therapies.

In HCC, glypican-3 (GPC3) has emerged as one of the most promising therapeutic targets (14). GPC3 is a membrane-associated heparan sulfate proteoglycan with minimal expression in normal adult tissues but aberrant re-expression in a subset of malignancies, most notably HCC (15). This tumor-restricted expression pattern, combined with low baseline tissue expression, makes GPC3 an attractive immunotherapeutic target (14, 15). Accordingly, GPC3-directed monoclonal antibodies, CAR-T cells, and BiTEs have demonstrated encouraging preclinical efficacy (16), and multiple clinical trials are ongoing in patients with HCC (e.g., NCT02748837, NCT04076137, and NCT01507168).

However, a major barrier to the clinical success of GPC3-targeted therapies is the pronounced heterogeneity of GPC3 expression in HCC. Both inter- and intra-tumoral variability have been widely documented, ranging from strong and uniform membrane expression to weak, patchy, or complete absence of GPC3 expression (17, 18). This heterogeneity significantly restricts patient eligibility and limits therapeutic efficacy, as CAR-T cells and BiTEs require sufficient antigen density for optimal T-cell engagement and cytotoxic activity (19). Consequently, antigen heterogeneity remains a central obstacle to durable and broadly effective GPC3-directed immunotherapy.

BiTEs are engineered fusion proteins composed of two single-chain variable fragments (scFvs), one targeting CD3 on T cells and the other recognizing a tumor-associated antigen (8). By physically linking T cells to cancer cells, BiTEs induce potent T-cell activation and cytotoxicity independent of co-stimulatory signaling (20). Blinatumomab, the first FDA-approved BiTE, has demonstrated transformative efficacy in B-cell malignancies (11). However, similar to CAR-T cell therapies, the application of BiTEs in solid tumors has been limited by the absence of uniformly expressed target antigens and the immunosuppressive tumor microenvironment.

Among the most clinically advanced GPC3-targeted cellular therapies is ECT204, developed using the ARTEMIS^®^ platform. This system employs an antibody–TCR (AbTCR) fusion that integrates antibody-based antigen recognition with the effector functions of a human γδ T-cell receptor (21). Unlike conventional CAR-T cells, ARTEMIS T cells engage the endogenous CD3 signaling complex, leveraging native TCR signaling pathways. This design has been associated with reduced cytokine hyperactivation and a lower risk of cytokine release syndrome and neurotoxicity, enabling the administration of higher therapeutic cell doses (22). ECT204 has completed a phase I clinical study in patients with HCC and has progressed into a Phase II clinical trial (ARYA-3, NCT04864054). Despite its favorable safety profile and clinical advancement, its therapeutic efficacy remains constrained by heterogeneous and often low-level GPC3 expression in tumor lesions.

Oncolytic viruses (OVs) offer a complementary strategy to overcome antigen heterogeneity by functioning as tumor-selective gene delivery platforms that can increase tumor antigen density while simultaneously remodeling the immunosuppressive tumor microenvironment. These engineered viruses preferentially replicate in tumor cells due to defects in antiviral pathways, including impaired interferon signaling, resulting in selective tumor lysis while sparing normal tissues (23). In addition to direct oncolysis, OVs induce immunogenic cell death, promote inflammatory cytokine release, and enhance immune cell infiltration into otherwise “cold” tumor microenvironments (24).

We previously developed CF33, a chimeric oncolytic poxvirus generated through recombination of multiple vaccinia strains (25). CF33 exhibits enhanced oncolytic potency compared to parental vaccinia backbones and is currently being evaluated in multiple phase I clinical trials. Building upon this platform, we engineered CF33-GPC3 as a tumor-selective antigen delivery system encoding GPC3 under a synthetic early promoter to enable robust antigen expression prior to viral cytolysis. We hypothesized that CF33-GPC3 could convert GPC3-negative or heterogeneous HCC tumors into uniformly GPC3-positive lesions, thereby broadening the applicability of GPC3-directed therapies, including ECT204, BiTEs, and monoclonal antibodies.

Here, we demonstrate that CF33-GPC3 induces robust GPC3 expression in tumor cells both *in vitro* and *in vivo*, achieving surface antigen levels comparable to those observed in endogenously GPC3-high HCC cells. Furthermore, we show that viral antigen delivery restores susceptibility of GPC3-negative tumors to GPC3- targeted immunotherapies, including ECT204, a GPC3-specific BiTE, and the monoclonal antibody codrituzumab. Collectively, these findings establish CF33-GPC3 as a novel strategy to overcome antigen heterogeneity and significantly expand the therapeutic landscape of GPC3-directed immunotherapy in HCC.

## Material & methods

### Study design

This study used an oncolytic virus to deliver GPC3 to tumor cells for subsequent targeting of those cells with GPC3-directed therapeutics. *In vitro* experiments were performed in duplicate or triplicate and repeated independently 2-3 times. *In vivo* studies utilized NSG mice aged 6–8 weeks; three animals per treatment group were used for immunohistochemistry staining (for negative control i.e. PBS, one mouse was used per time point). Seven to ten mice per group were used for therapeutic experiments, ensuring adequate statistical power and balanced tumor sizes at treatment initiation. Prior to treatment initiation, mice were re-grouped based on their tumor volume so as to achieve similar average tumor volume in each treatment group.

Animals were excluded from analysis if they died within 1 week of virus injection. Animal health was monitored daily by the Department of Comparative Medicine at City of Hope, and humane endpoints were applied according to American Veterinary Medical Association guidelines. Survival monitoring was not blinded. All animal experiments were conducted under protocols approved by the Institutional Animal Care and Use Committee, and all procedures adhered to institutional review board policies.

### Cell lines and virus

CV-1 (African green monkey kidney), HepG2 and SK-Hep1 (liver cancer), SNU-16 (gastric cancer) and As-PC-1 (pancreatic cancer) were obtained from ATCC. HepG2GPC3-KO cells were generated by CRISPR/Cas9-mediated knockout of GPC3 in HepG2 cells. CV-1 cells were grown in DMEM; cancer lines were grown in RPMI-1640 with 10% FBS, 2 mmol/L L-glutamine, and 100 U/mL penicillin–streptomycin (Corning). All cultures were maintained at 37 °C, 5% CO_2_.

CF33, a chimeric poxvirus generated from nine parental strains, was engineered as described previously (25). Briefly, to generate CF33-GPC3, the viral J2R gene was replaced with a GPC3 cassette, encoding a non-functional human GPC3, via homologous recombination in CV-1 cells. Recombinants were plaque-purified under drug selection and confirmed by PCR and PacBio sequencing.

### Generation of GPC3 ARTEMIS T cells (ECT204)

GPC3 antibody sequences were engineered and engrafted into ARTEMIS constructs containing AbTCR and costimulatory receptor recognizing extracellular domains of GPC3. Constructs were cloned into pCDH lentiviral vectors and used to transduce CD3/CD28-activated primary human T cells. Transduction efficiency was assessed by flow cytometry, showing ~30% AbTCR-positive cells (**Supplementary Figure 1**).

### Bioinformatic analyses

Transcriptomics data from the cancer genome atlas (TCGA, https://portal.gdc.cancer.gov) for hepatocellular carcinoma cohort (374 tumor samples and 50 adjacent tissue samples) were retrieved using the TCGAbiolinks R package (v 2.32.0) (26). Differential expression analysis was conducted using DESeq2 (v 1.44.0) (27). To identify significantly differentially expressed genes, a threshold of log_2_ fold change greater than 1 and an adjusted p-value less than 0.05 was applied. Differentially expressed genes visualized on a volcano plot using ggplot2 (v 3.5.2). Normalized counts for GPC3 were extracted from the DESeq2 output and plotted across tumor and normal samples using ggplot2 (v 3.5.2). A t-test was used to assess statistical significance in GPC3 expression between conditions.

Proteomics data for hepatocellular carcinoma were obtained from the national cancer institute Proteomics Data Commons (https://proteomic.datacommons.cancer.gov/pdc/, PDC Study Identifier: PDC000198). The dataset includes paired tumor and adjacent liver tissue samples from 165 hepatocellular carcinoma patients analyzed using tandem mass tag (TMT) 11-plex labeling followed by nanoLC-MS/MS. Note that all HCC cases in this dataset are HBV-related. Protein abundance for GPC3 was extracted and visualized using ggplot2 (v 3.5.2), and statistical significance between paired tumor and normal samples was assessed using a paired t-test.

### Cytotoxicity and virus growth kinetics

Target cells (20, 000–60, 000/well) were infected with virus, then co-cultured with mock or ECT204 T cells at 1:1 ratio. Cell survival was assessed at 24–72 h by LDH release (CyQUANT LDH assay kit; Cat# C20301; ThermoFisher) or luciferase-based assays.

For virus growth kinetics analysis, cancer cells were infected at MOI 0.01, lysates were collected at 24, 48, and 72 h, and titers quantified by standard plaque assay on CV-1 cells as previously described (25).

### GPC3-specific BiTE

A BiTE was designed by fusing scFvs of anti-GPC3 (sequence obtained from US7919086B2) and anti-CD3 (OKT3) with glycine–serine linkers, cloned into an expression plasmid, and transiently expressed in CHO cells. BiTEs were purified by nickel affinity and validated for binding by flow cytometry.

### Flow cytometry

For virus-induced GPC3 expression, infected cells were stained with Zombie UV fixable dye and Alexa Fluor 488–anti-GPC3 (Cat#NBP-2-44486; Novus Biologicals), then analyzed on a BD Fortessa. To determine activation status of immune cells, cancer cells were plated in a round-bottom 96-well plate. Virus was added to the cells 1 hour later at different MOIs. Immune cells (CAR-Ts or NK cells) were added to the wells 3 hours post-infection and plates were incubated for 24- or 48-hour. To stain for CD107 (de-granulation marker), PE-conjugated CD107 antibody was added to the co-culture at the time of adding immune cells. At 24- or 48-h post-infection, cells were harvested by trypsinization and stained with Zombie UV fixable dye (Cat#423108) followed by staining with markers for different immune cells and their activation markers. The antibodies used are: anti-CD45-APC (clone HI30), anti-CD3-PE/Cy7 (Clone SK7), anti-CD8-AF700 (Clone SK1), anti-IFNy-AF488 (clone 4S.B3), anti-CD69-APC/Cy7 (clone FN50), anti-CD137-Percp/Cy5.5 (clone 4B4-1). All antibodies were purchased from BioLegend Inc.

### Quantification of surface GPC3 molecules

Surface GPC3 copy number was quantified using Quantum™ Alexa Fluor^®^ 488 MESF beads (Cat# 488A; Bangs Laboratories) according to the manufacturer’s instructions. Briefly, SK-Hep1 and HepG2^GPC3-KO^ cells were either mock-infected or infected with CF33-GPC3 at an MOI of 0.1, while parental HepG2 cells were mock-infected and used as a positive control. Eighteen hours post-infection, cells were harvested and stained with Zombie Live/Dead Fixable dye, followed by Alexa Fluor^®^ 488-conjugated anti-GPC3 antibody. Samples were analyzed on a BD Fortessa flow cytometer. Quantum™ Alexa Fluor^®^ 488 MESF calibration beads, containing defined numbers of Alexa Fluor^®^ 488 molecules per bead population, were acquired using the same instrument settings. A calibration curve was generated from the MESF bead populations, and the number of GPC3 molecules per cell was calculated by converting the geometric mean fluorescence intensity (MFI) of stained cells to molecules of equivalent soluble fluorochrome (MESF) values using the manufacturer provided analysis spreadsheet. Assuming a 1:1 fluorophore-to-antibody labeling ratio and one antibody molecule bound per GPC3 molecule, MESF values were converted to the estimated number of GPC3 molecules expressed on the cell surface.

### Immunofluorescence

SK-Hep1 cells were infected with CF33ΔJ2R or CF33-GPC3 (MOI 0.1), fixed, permeabilized, and stained with anti-GPC3 and anti-vaccinia antibodies, followed by Alexa Fluor-488 and PE-conjugated secondary antibodies. DAPI counterstain was used, and images acquired with EVOS FL Auto.

### Immunogenic cell death

Immunogenic cell death (ICD) was evaluated using three established markers: cell-surface exposure of calreticulin, extracellular release of ATP, and release of high-mobility group box 1 (HMGB1). Calreticulin exposure was assessed by flow cytometry. Briefly, mock- or CF33-GPC3–infected cells were harvested approximately 24 hours post-infection using 10 mmol/L EDTA, washed, and stained with Zombie UV Live/Dead Fixable dye (Cat#423107; BioLegend Inc.), followed by staining with AlexaFluor 488–conjugated anti-calreticulin antibody (ab196158; Abcam) or the corresponding isotype control (ab199091; Abcam). Cells were subsequently fixed with 4% paraformaldehyde and analyzed using a BD LSRFortessa cytometer. Calreticulin levels were analyzed on live cells.

Extracellular ATP release was quantified in the culture supernatants (24 hours post-infection) using a luminescence-based ATP determination kit (A22066; Molecular Probes), according to the manufacturer’s instructions. HMGB1 release was assessed by Western blot analysis using a fixed volume (30 µL) of supernatant collected from mock- or virus-infected cells. HMGB1 was detected using a rabbit anti-HMGB1 antibody (ab18256; Abcam), followed by a donkey anti-rabbit secondary antibody (Cat# ab205718; Abcam).

### Immunohistochemistry

Tumors were FFPE processed and stained on a Ventana automated stainer. Antibodies included anti-CD3, anti-GPC3 and anti-vaccinia virus. Slides were scanned with NanoZoomer S360.

The biopsy samples stained for GPC3 (**Figure 1D**) were obtained from 9 individuals with confirmed HCC. The slide (MTB80) was generated by City of Hope’s pathology core many years ago and has since been routinely used as a positive/negative control for GPC3 staining. As archival, de-identified material utilized for assay control, these samples did not require IRB approval.

**Figure 1:**
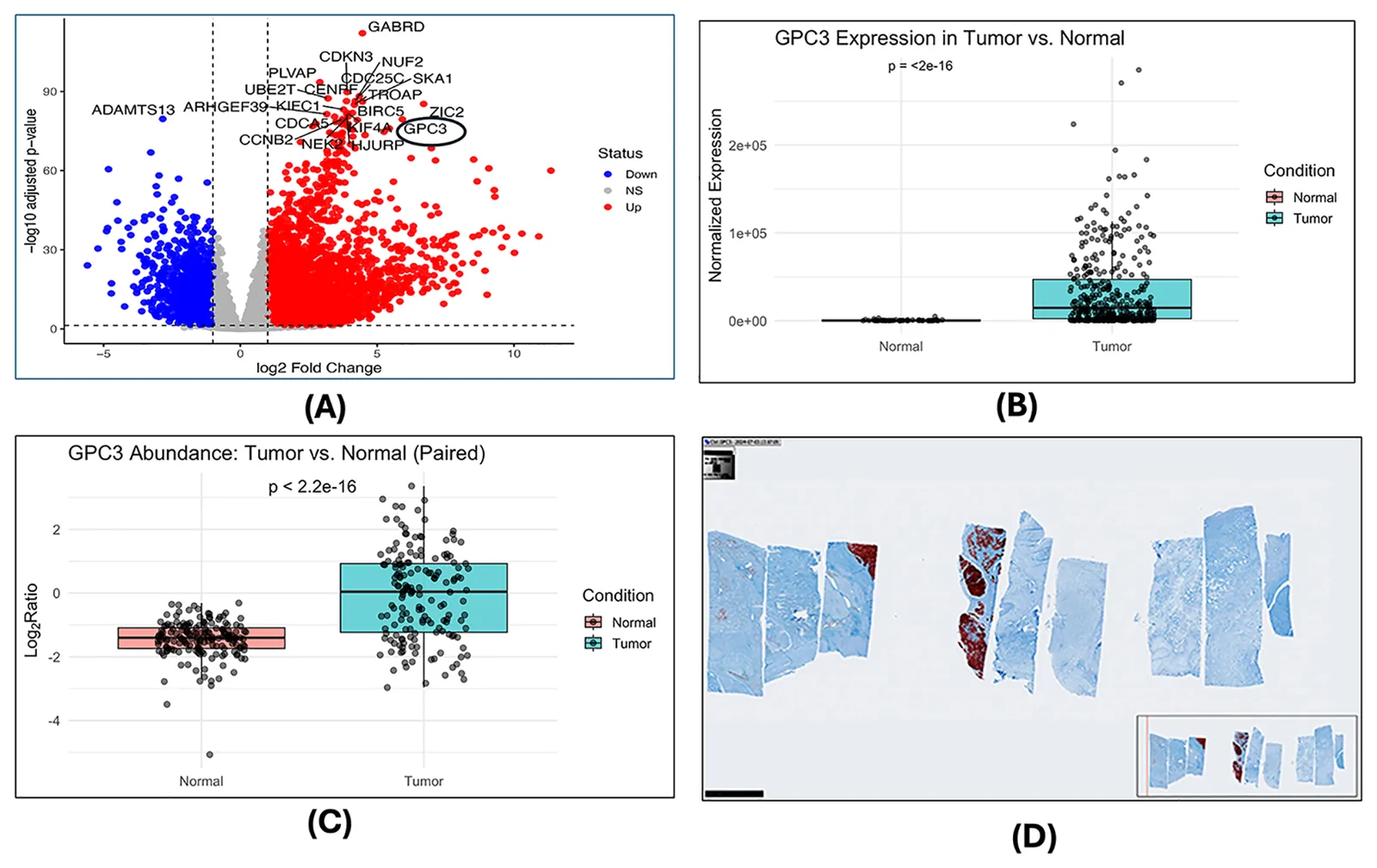
Heterogeneous expression of GPC3 in HCC. **(A)** Heatmap of the top 20 differentially expressed genes in TCGA-HCC shows GPC3 upregulation in HCC samples compared to normal tissues. **(B)** GPC3 transcript levels (normalized counts) in TCGA-HCC reveal subsets of tumors with low or absent expression (n=374 for HCC and 50 for Normal). Statistical comparison was performed using student’s *t*-test. **(C)** Proteomic data from 165 paired HCC and adjacent liver samples (NCI proteomics data; PDC000198) show variable GPC3 protein abundance. Protein abundance is expressed as log2ratio of the samples compared to a reference. Statistical comparison was performed using student’s *t*-test. **(D)** GPC3 staining on biopsy samples from 9 HCC patients (MTB80).

### Tumor model and treatment

All animal studies were conducted under City of Hope Institutional Animal Care and Use Committee approved protocol (IACUC#15003) in compliance with NIH’s guideline for the use of laboratory animals. Female NOD SCID Gamma (NSG) mice, 6–8 weeks of age, were obtained from the Jackson Laboratory and housed in pathogen-free, HEPA-filtered ventilated cages within a biosafety level 2 containment suite at the City of Hope animal facility. Mice were acclimated for 1 week prior to experimental procedures. Mice were implanted with 5×10^6^ HepG2^GPC3-KO^ cells in Matrigel/PBS. Once tumors reached 100–200 mm³, mice were randomized (7–10 mice/group) and treated with PBS (negative control), ECT204, OV+mock T cells, or OV+ECT204. Treatments (1×10^6^ PFU OV or 5×10^6^ T cells) were delivered intratumorally (IT) or intravenously (IV). Tumor volumes were monitored biweekly; mice were euthanized if body weight loss exceeded 20% or tumors exceeded 1, 500 mm³ in volume. Monitoring of mice and tumor measurement were not blinded. Mice were euthanized by gradual-fill carbon dioxide (CO_2_) inhalation at 40% chamber volume/min in accordance with institutional animal care and use guidelines.

### Statistical analyses

Data are presented as mean ± SD unless otherwise indicated. For tumor regression studies, 7–10 mice/group were used based on established standards in the field and were not pre-determined using formal statistical power calculations. Statistical analyses were performed using unpaired two-tailed Student’s t tests or ANOVA, as appropriate, with specific tests detailed in the corresponding figure legends. A 95% confidence interval was used to assess statistical significance, and P < 0.05 was considered statistically significant. All analyses were conducted using GraphPad Prism version 8.

## Results

### Expression of GPC3 is highly heterogeneous in HCC

GPC3 is an oncofetal protein expressed during fetal development but largely absent in adult tissues. In HCC and certain other malignancies, however, GPC3 is re-expressed and ranks among the most highly upregulated genes in HCC (15). To further validate GPC3 expression, we analyzed transcriptomic data from The Cancer Genome Atlas (TCGA, https://portal.gdc.cancer.gov). Among the top 20 differentially expressed genes between normal tissue and HCC tumors, GPC3 was prominently represented (**Figure 1A**). While HCC tumors exhibited elevated GPC3 expression, a substantial subset displayed levels comparable to that in normal tissues (**Figure 1B**). We further examined GPC3 protein abundance using a dataset from the National Cancer Institute Proteomics Data Commons (https://proteomic.datacommons.cancer.gov/pdc/PDC Study Identifier: PDC000198), which included paired tumor and adjacent liver samples from 165 HCC patients. Consistent with the transcriptomic findings, GPC3 protein levels showed considerable variability, with nearly half of the tumor samples exhibiting expression comparable to normal tissue (**Figure 1C**). To further validate these observations, we analyzed immunohistochemical (IHC) staining of biopsy samples from 9 HCC patients. Again, marked heterogeneity was evident: 2 samples were strongly positive, 3 were weakly positive, and 4 were negative for GPC3 expression (**Figure 1D**). Collectively, these data show that while GPC3 is a tumor-associated marker in HCC, its expression is highly heterogeneous in HCC patients.

### CF33-GPC3 effectively converts GPC3-negative cancer cells into GPC3-positive *in vitro* and *in vivo*

The oncolytic virus CF33-GPC3 was engineered by inserting a GPC3 expression cassette into the J2R locus of the chimeric poxvirus CF33. We first validated transgene expression by RT-PCR and Western blotting, confirming GPC3 expression within 24 hours at levels comparable to GAPDH (**Supplementary Figure 2**). To assess surface expression of GPC3, we utilized the GPC3-knockout HepG2 line (HepG2^GPC3-KO^) and the innately GPC3-negative HCC line SK-Hep1. Flow cytometry showed efficient induction of GPC3 in both cell lines: SK-Hep1 cells were uniformly GPC3-positive within 24 hours at MOI 1, whereas HepG2^GPC3-KO^ cells exhibited complete expression by 48 hours (**Figures 2A, B**). Similar induction was observed in other cell lines representative of different tumor types, including cholangiocarcinoma, pancreatic, and gastric cancer lines (**Supplementary Figure 3**).

**Figure 2:**
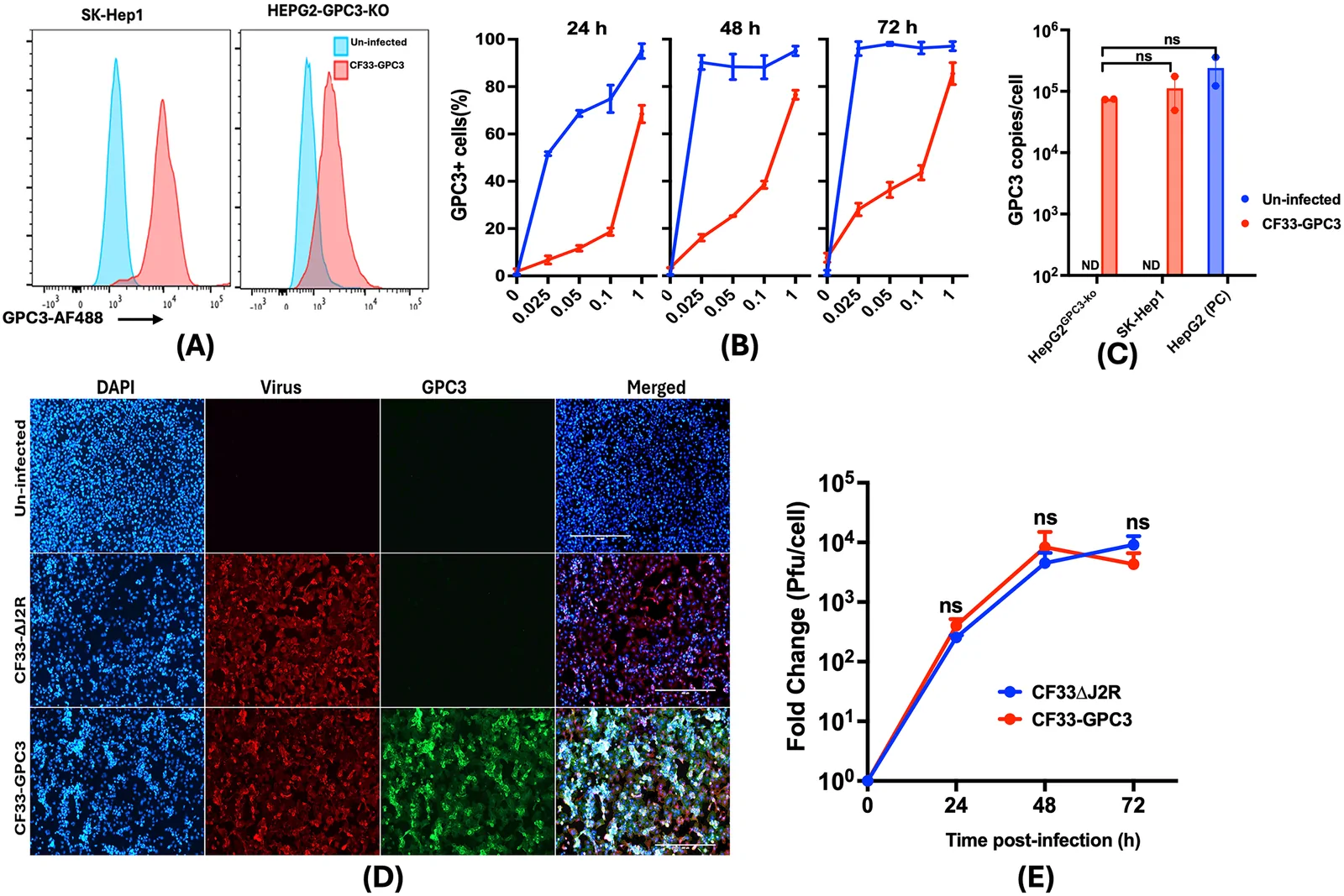
OV-mediated robust surface expression of GPC3 *in vitro*. **(A)** Flow cytometric analysis of GPC3 expression in SK-Hep1 and HepG2^GPC3-KO^ cells infected with CF33-GPC3. **(B)** GPC3 expression on cell surface of SK-Hep1 and HepG2^GPC3-KO^ cells at indicated MOIs and time points. Data presented as mean ± SEM from 3 independent experiments. **(C)** Quantification of surface GPC3 molecules using pre-labelled calibration beads. Data presented as mean ± SEM from 2 independent experiments. **(D)** Immunofluorescence analysis confirmed GPC3 expression only in CF33-GPC3–infected SK-Hep1 cells, with viral infection evident in both CF33-GPC3 and CF33ΔJ2R infected cells. Scale bar= 400 µm. **(E)** Comparison of growth kinetics of CF33-GPC3 and CF33ΔJ2R. The oncolytic virus CF33-GPC3 has similar growth kinetics as the control virus CF33ΔJ2R in HepG2 cells. Data presented as mean ± SEM from 2 independent experiments. Statistical analyses in **(C, E)** were performed using 2way ANOVA; ns, not significant.

To quantify antigen density, we used calibration beads labeled with defined fluorophore equivalents. GPC3 was undetectable in uninfected HepG2^GPC3-KO^ or SK-Hep1 cells, but within 18 hours of CF33-GPC3 infection both cell types expressed GPC3 molecules at levels comparable to that in native GPC3-high HepG2 cells (**Figure 2C**).

We also used immunofluorescence to visually confirm surface expression of GPC3 in OV-infected cells. SK-Hep1 cells infected with either control virus (CF33ΔJ2R) or CF33-GPC3 were stained for viral antigen, GPC3, and counterstained with DAPI. Viral antigen was detectable in both groups, but GPC3 expression was exclusive to CF33-GPC3–infected cells. Notably, even at a low MOI of 0.1, nearly all cells expressed GPC3 by 24 hours, underscoring the efficiency of transgene delivery (**Figure 2D**).

In order to determine if the insertion of the GPC3 expression cassette in the virus genome has any negative effect on the ability of the virus to replicate in cancer cells, we compared replication kinetics of CF33-GPC3 and CF33ΔJ2R in HepG2 cells. Viral titers were measured by plaque assay at different time points following infection. Both viruses demonstrated similar replication kinetics, confirming that GPC3 insertion does not attenuate virus replication (**Figure 2E**). We next evaluated transgene delivery *in vivo*. Subcutaneous HepG2^GPC3-KO^ tumors were established in NSG mice and treated with a single intratumoral (IT) or intravenous (IV) dose of PBS or CF33-GPC3 once tumors reached ~100–200 mm³. Tumors harvested on days 3 and 7 post-treatment, were analyzed for GPC3 expression by immunohistochemistry. As expected, PBS-treated tumors were negative for GPC3, whereas CF33-GPC3–treated tumors expressed GPC3 at both time points. Expression was higher at day 7 than at day 3, independent of delivery route. While IT injection yielded stronger expression at day 3, IV and IT delivery achieved comparable levels by day 7 (**Figure 3**).

**Figure 3:**
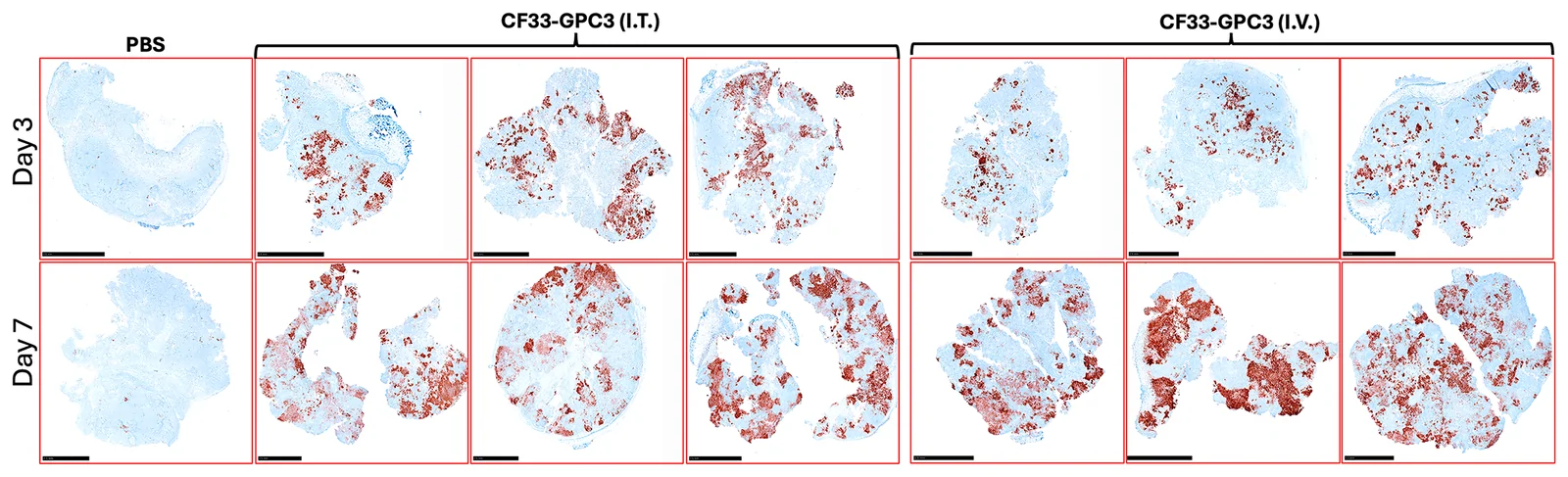
OV-mediated GPC3 delivery to GPC3-negative tumors *in vivo*. NSG mice bearing subcutaneous HepG2^GPC3-KO^ tumors were treated with CF33-GPC3 via IT or IV injection (1E06 PFU/tumor for IT and 2E06 PFU/mouse for IV). Tumors harvested on days 3 and 7 were assessed by IHC for GPC3 expression. Each tumor section is from an individual mouse (n=1 for PBS and n=3 for other treatments at each time point). Scale bar=2.5 mm.

### OV-delivered GPC3 enables killing of cancer cells by ECT204 (anti-GPC3-AbTCR cells)

After confirming the ability of the virus to efficiently deliver GPC3 to cancer cells, we assessed if the virus-encoded GPC3 on cell surface can be recognized by the GPC3-targted T cells *i.e*. ECT204. For this, HepG2^GPC3-KO^ and SK-Hep1cells were infected with the OV at varying MOIs and co-cultured with either mock-transduced T cells or ECT204 at a 1:1 ratio for 24 or 48 hours. Following co-culture, flow cytometry was used to assess T cell activation and activity. Surface expressions of CD137 (4-1BB) and CD69 were considered as activation markers, and surface expression of CD107 as well as intracellular interferon-γ (IFNγ) were used as indicators of T cell activity. Flow cytometry data demonstrated that ECT204 cells were activated by OV-infected cancer cells in an MOI-dependent manner (**Figures 4A, B**).

**Figure 4:**
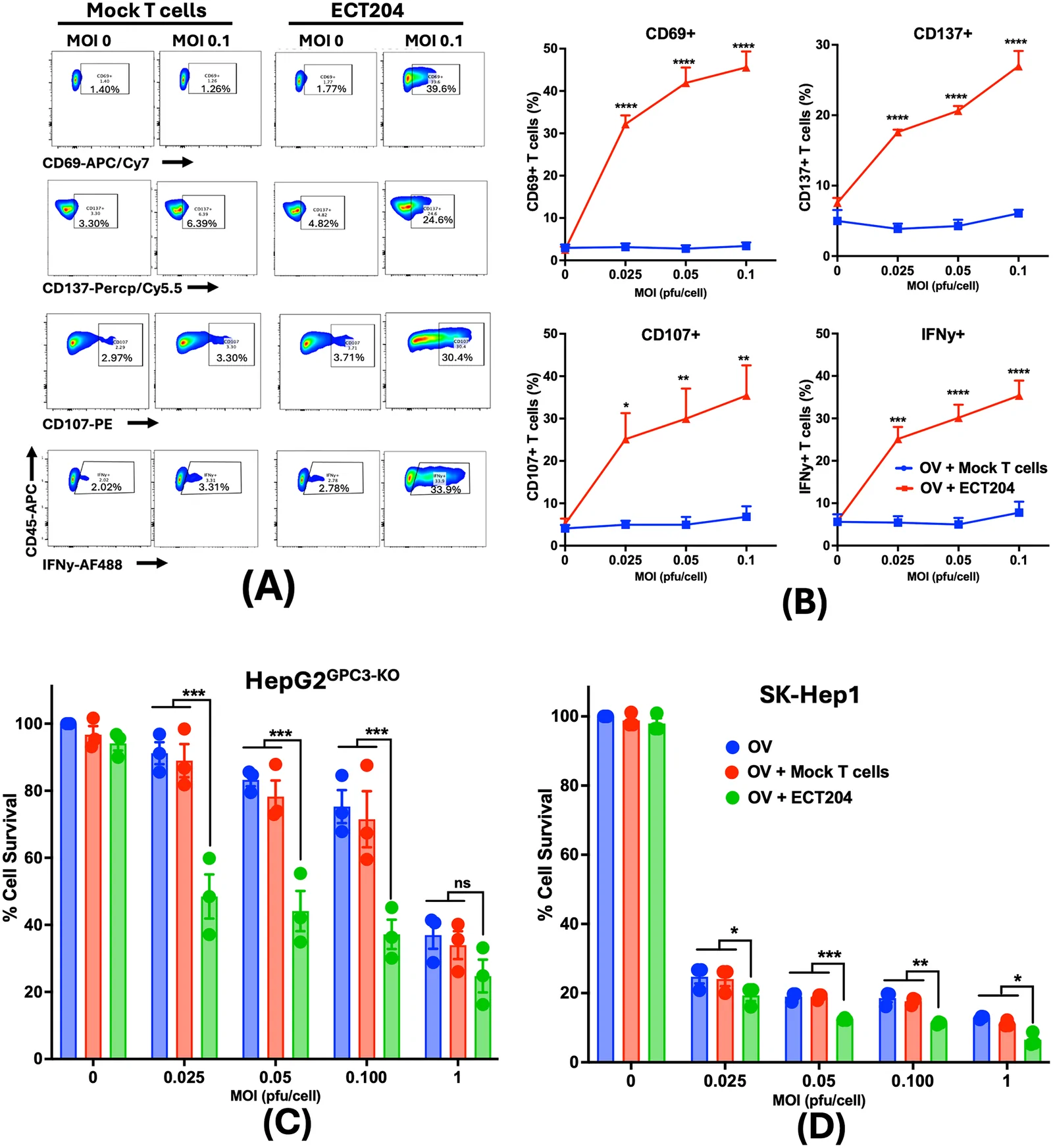
OV-delivered GPC3 activates ECT204 cells and enables them to kill cancer cells *in vitro*: **(A, B)** Activation of ECT204 by OV-delivered GPC3. HepG2^GPC3-KO^ cells were infected with OV (CF33-GPC3) at varying MOIs and co-cultured with mock T cells or ECT204 T cells at a 1:1 ratio for 24–48 h **(A)** T cell activation (CD137, CD69) and activity (CD107, IFNγ) were assessed by flow cytometry. **(B)** OV-infected cancer cells induced MOI-dependent activation of ECT204 cells. **(C, D)** Cytotoxicity assays using HepG2^GPC3-KO^ and SK-Hep1 cells showed significantly greater killing with OV plus ECT204 T cells compared to OV alone or OV plus mock T cells in HepG2^GPC3-KO^ **(C)** and SK-Hep1 **(D)** cells at 48 hours post-infection. Data in **(A)** is representative of 3 independent experiments and **(B–D)** are plotted as mean ± SEM from 3 independent experiments. Statistical comparison was performed using 2way ANOVA; *p < 0.05; **p < 0.01; ***p < 0.005; ****p < 0.001; ns=not significant.

To assess whether the activation of ECT204 cells by virus-encoded GPC3 translated into cytotoxicity against OV-infected cancer cells, we performed *in vitro* cytotoxicity assays in HepG2^GPC3-KO^ and SK-Hep1cells. In both cell lines, compared to the OV alone or OV combined with mock T cells, the combination of OV and ECT204 led to significantly greater cancer cell killing. The HepG2^GPC3-KO^ cell line appeared less sensitive to virus-mediated killing, resulting in a more pronounced difference in cytotoxicity between OV alone and OV plus ECT204 cells (**Figure 4C**). In contrast, SK-Hep1 cells were highly sensitive to the virus, and although differences in killing were observed between OV alone and OV plus ECT204, they were comparatively smaller (**Figure 4D**). Nevertheless, across both cell lines and MOIs tested, the combination of OV and ECT204 cells consistently resulted in significantly enhanced cytotoxicity. We have also tested the combination of the OV and ECT204 *in vitro* using cell lines representative of other tumor types including pancreatic cancer and gastric cancer. Interestingly, the combination seems to work even better in these non-HCC tumor types (**Supplementary Figure 4**).

### Combination of OV with ECT204 enhances anti-tumor efficacy in liver cancer xenograft models

We next evaluated whether OV-mediated antigen delivery could functionally expand the therapeutic activity of ECT204 *in vivo* using NSG mice bearing subcutaneous HepG2^GPC3-KO^ xenografts. In the first study, treatments were delivered intratumorally once tumors reached ~100–200 mm³. Mice were randomized to receive PBS, ECT204 cells, OV plus mock T cells, or OV plus ECT204 cells. A single dose of OV (1×10^6^ PFU) was administered, followed one week later by T cells (5×10^6^) (**Figure 5A**). Tumor volumes were measured twice weekly (**Figure 5B**). ECT204 alone did not impact tumor growth, with kinetics comparable to PBS-treated controls. As expected, OV in presence of mock T cells induced tumor growth inhibition compared with PBS or ECT204 treatment. Notably, the OV plus ECT204 combination conferred superior tumor control compared to OV plus mock T cells, achieving statistically significant improvement.

**Figure 5:**
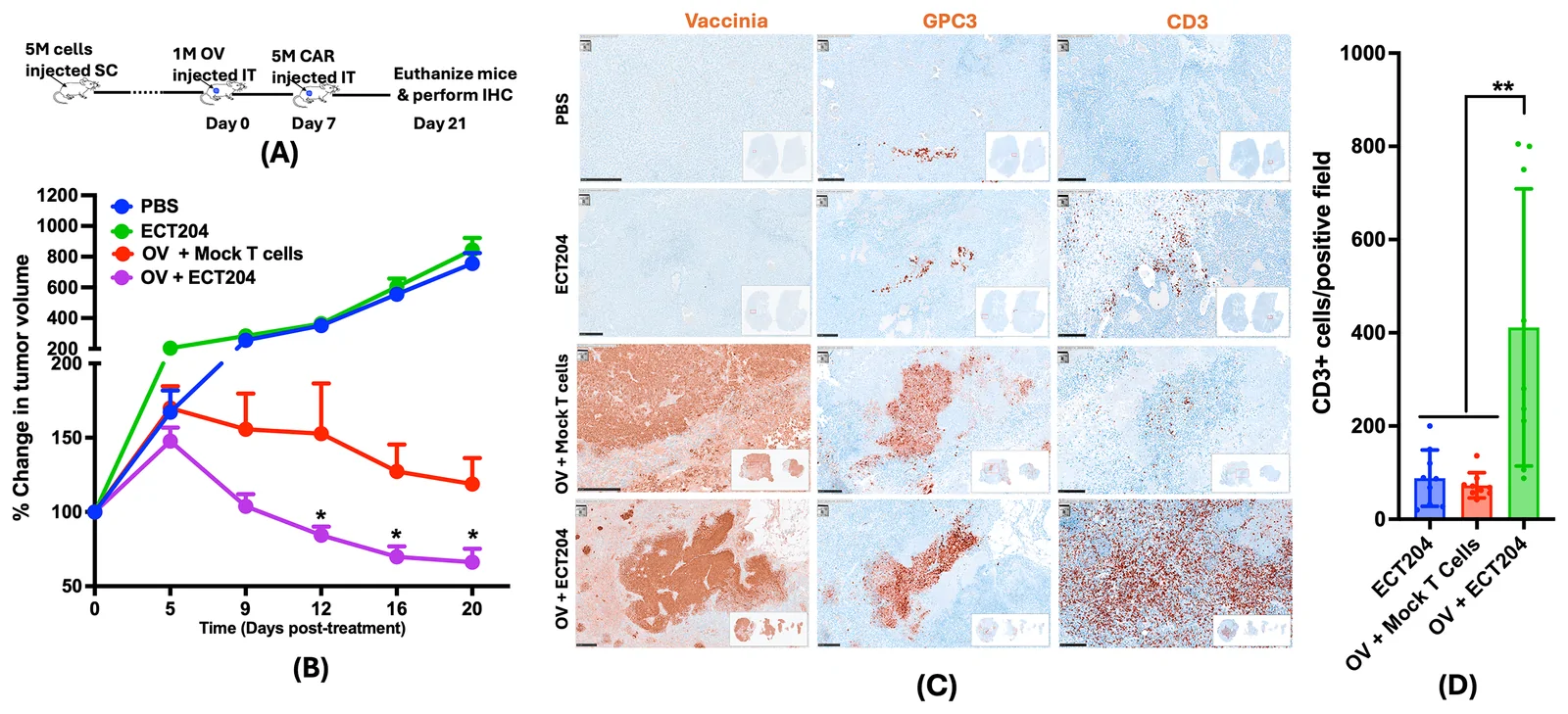
Combination of IT delivered OV and ECT204 results in improved anti-tumor efficacy in HepG2^GPC3-KO^ xenograft model. **(A)** NSG mice bearing subcutaneous HepG2^GPC3-KO^ tumors were treated as depicted (n=7 mice for ECT204, n=8 for all other groups). **(B)** Tumor volumes were measured twice weekly and average tumor volume ± SEM for each group was plotted and compared. Statistical analysis was performed using 2way ANOVA comparing all treatment groups at each time point. P values on the graph indicate differences between OV + mock vs OV + ECT204. **(C)** Tumors were harvested at the end of the study and IHC was performed to visualize virus infected cells, GPC3 expression and T cells distribution. Scale bar= 250 µm. **(D)** CD3+ cells (T cells) were counted from 3 tumors/group (3 positive fields/tumor counted; plotted as mean ± SD) and compared among the treatment groups. Statistical analysis using one-way ANOVA; *p<0.05, **p < 0.01.

At study endpoint, tumors were collected for immunohistochemistry. Anti-virus staining confirmed widespread OV distribution in all OV-treated tumors (**Figure 5C**). GPC3 expression was observed in viable tumor regions of OV-injected groups, whereas necrotic regions, despite being virus-positive, were GPC3-negative. T cell staining revealed limited persistence of mock T cells, while ECT204 cells localized predominantly within viable tumor regions. Importantly, ECT204 cells demonstrated higher intratumoral density in mice receiving OV, suggesting enhanced infiltration and expansion in response to virus-encoded GPC3 (**Figure 5D**).

In a second *in vivo* study, both OV and T cells were delivered intravenously. Consistent with the intratumoral model, OV in presence of mock T cells resulted in tumor growth inhibition compared with PBS or ECT204 (**Figure 6A**). However, OV plus ECT204 therapy produced significantly greater tumor control than OV plus mock T cells. Endpoint histopathology by hematoxylin and eosin staining further demonstrated limited necrosis in tumors treated with PBS or ECT204 alone, whereas tumors from OV-treated groups exhibited extensive necrotic regions (**Figure 6B**). Tumors treated with OV plus ECT204 were largely necrotic, consistent with enhanced cytotoxicity. Notably, in this xenograft model, residual necrotic tumor mass persisted despite almost complete loss of viable tumor cells, indicating that caliper-based volume measurements may underestimate therapeutic efficacy in this model.

**Figure 6:**
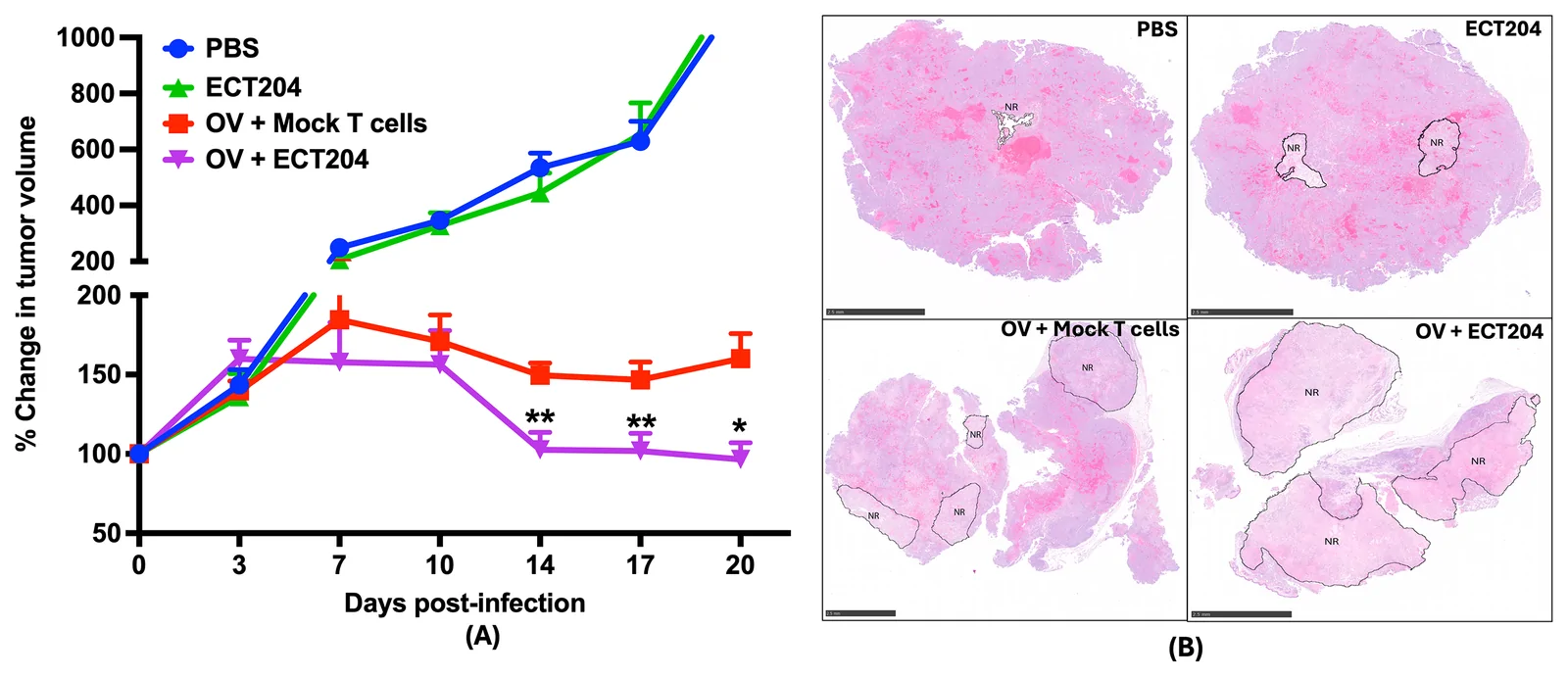
Combination of IV delivered OV and ECT204 results in improved anti-tumor efficacy in HepG2^GPC3-KO^ xenograft model. **(A)** Mice (n=10 for OV + ECT204 and n=7 for all other groups) were treated as in **Figure 5A** except that all treatments were given through IV route. Tumor volumes were measured twice weekly and average tumor volume ± SEM for each group was plotted and compared. Statistical analysis was performed using 2way ANOVA comparing all treatment groups at each time point. P values on the graph indicate differences between OV + mock vs OV + ECT204; *p < 0.05. **(B)** Representative H&E staining of tumors at study endpoint. There were minimal necrotic regions in the PBS or ECT204 treated tumors, tumors treated with OV plus mock T cells displayed increase in necrotic area whereas the tumors treated with the combination of OV and ECT204 were mostly necrotic (necrotic regions have been highlighted with black line; NR= necrotic region). Scale bar= 2.5 mm. **p<0.01.

Together, these findings demonstrate that OV-mediated antigen delivery can effectively sensitize antigen-deficient tumors to ECT204 therapy and substantially enhance the anti-tumor activity of this next-generation AbTCR platform *in vivo*.

### OV-delivered GPC3 promotes BiTE-mediated T cell activation and killing of tumor cells

We first assessed the ability of the BiTE to bind its intended targets. To do this, we conjugated the BiTE with a fluorescent dye (Alexa-647) and then incubated it with either HepG2 cells (GPC3+) or human T cells (CD3+), followed by flow cytometric analysis. As expected, the BiTE bound to both GPC3 and CD3 (**Figure 7A**).

**Figure 7:**
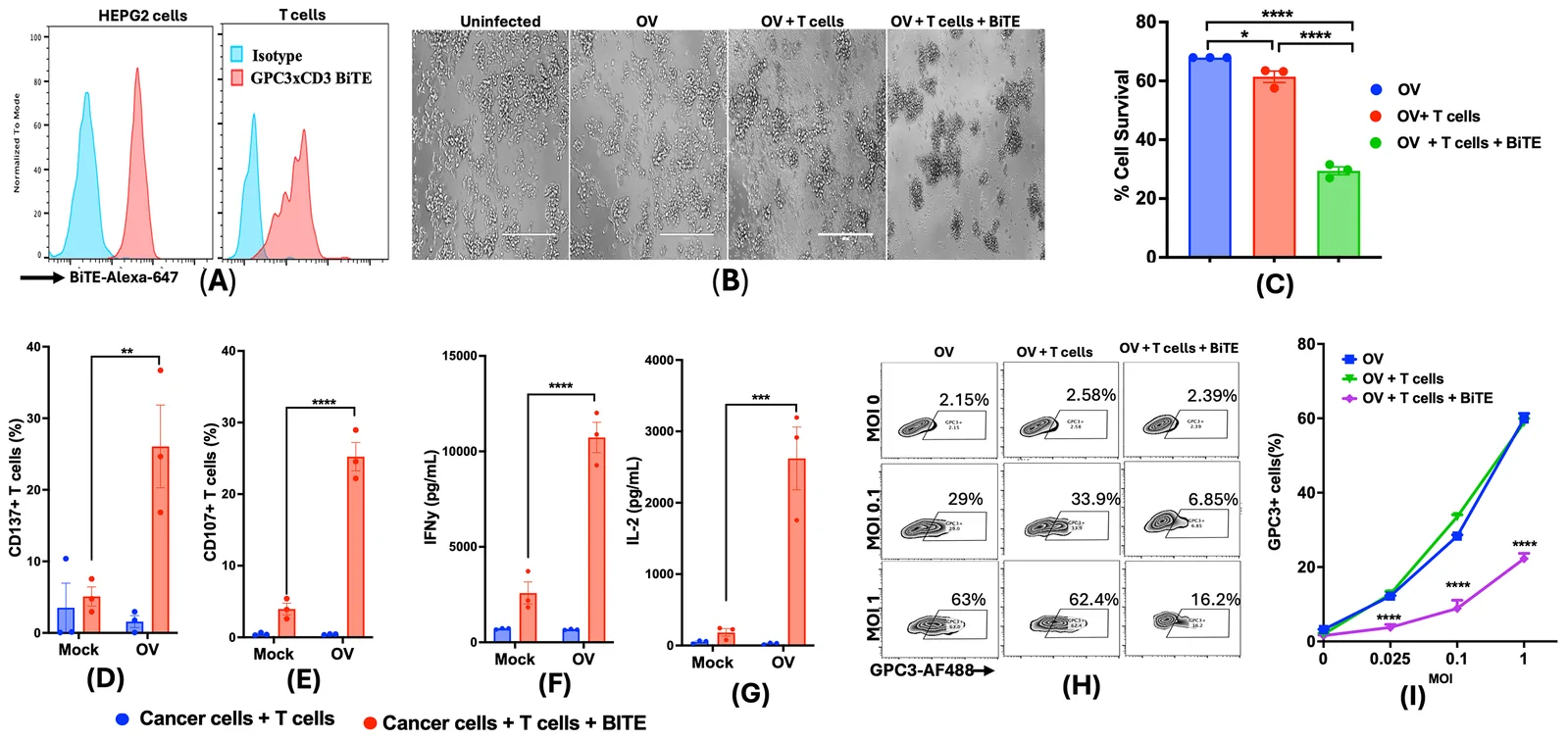
GPC3-targeted BiTE facilitates T cell activation and cytotoxicity against tumor cells expressing virally delivered GPC3. **(A)** Binding of Alexa-647–conjugated BiTE to GPC3 on HepG2 cells and to CD3 on primary human T cells was confirmed by flow cytometry. **(B)** HepG2^GPC3-KO^ cells were infected with OV (CF33-GPC3) at an MOI of 0.1 and co-cultured with human T cells (1:1) in the presence or absence of GPC3 BiTE (at 5 µM concentration). Representative images taken at 48 hours post-infection, show higher killing of OV-infected cancer cells by T cells in presence of BiTE. **(C)** Cytotoxicity assay was performed as in **(B)** using T cells from 3 healthy donors and cell survival was compared 48 hours post-treatment. Statistical analysis using one-way ANOVA; *p < 0.05, ****p < 0.001. **(D, E)** T cells activation markers (CD137 & CD107) were analyzed after the co-culture of T cells with mock-infected or OV-infected cancer cells in presence or absence of BiTE. **(F, G)** T cell activation-associated cytokines (IL-2 and IFNγ) were measured in the supernatant of the co-culture, using ELISA. **(H)** Twenty-four hours after co-culture, cells were analyzed by flowcytometry to determine proportion of GPC3+ cancer cells. BiTE treatment led to marked reduction in GPC3+ cells. **(I)** Pooled data from T cells of 3 independent donors, presented as mean ± SEM. For **(D–G, I)** statistical comparison was performed using 2way ANOVA; *p < 0.05, **p < 0.01, ***p < 0.005, ****p < 0.001. Data in **(A, B, H)** are shown for T cells from one of the 3 donors. Data in **(C-G)** are shown as mean ± SEM for T cells from 3 independent donors.

Next, we evaluated whether the BiTE could facilitate T cell-mediated killing of tumor cells displaying virus-encoded GPC3. HepG2^GPC3-KO^ cells were infected with CF33-GPC3 and co-cultured with freshly isolated human T cells in the presence or absence of the BiTE. After 48 hours, cell viability was assessed. The combination of OV and T cells in presence of BiTE led to significantly greater tumor cell killing compared to OV alone or OV plus T cells in the absence of BiTE. The cytotoxicity assay was performed using T cells from 3 healthy donors, and similar results were obtained (**Figures 7B, C**).

To assess T cell activation, we measured the surface expression of CD107 and CD137 as well as the secretion of the cytokines IL-2 and IFNγ. In the absence of BiTE, T cells remained mostly negative for the surface markers. Likewise, minimal CD107 and CD137 expressions were observed when BiTE was present without OV infection. However, significantly higher percentage of T cells expressed CD107 and CD137 when co-cultured with OV-infected tumor cells in the presence of BiTE (**Figures 7D, E**). Furthermore, secreted cytokines were either undetectable or very low when the T cells were co-cultured with OV-infected cells in the absence of BiTE or with un-infected cells in presence of BiTE, however, high levels of IL-2 and IFNγ were detected in the supernatant when the T cells were co-cultured with OV-infected cancer cells in presence of the BiTE (**Figures 7F, G**).

To assess whether BiTE-mediated tumor cell killing was dependent on GPC3 expression, we co-cultured T cells with either uninfected or OV–infected HepG2^GPC3-KO^ cells in the presence or absence of the BiTE. Next, GPC3 expression on target cells was quantified by flow cytometry at various time points. In the absence of BiTE, over 30% of cells remained GPC3-positive following infection with the OV at an MOI of 0.1, regardless of T cell co-culture. In contrast, only ~7% of cells were GPC3-positive when BiTE was included, indicating selective depletion of GPC3-positive cells. Likewise, at an MOI of 1, GPC3+ cells constituted less than 17% in the presence of BiTE compared to over 62% without BiTE (**Figure 7H**). These results suggest that BiTE promotes T cell–mediated killing of tumor cells displaying virally-encoded GPC3. Similar trends were observed across T cells from all 3 donors; pooled data are shown in **Figure 7I**.

### OV-delivered GPC3 promotes ADCC by NK cells in presence of GPC3-targeted antibody

Monoclonal antibodies (mAbs) represent a major class of approved cancer therapeutics, with one of their principal mechanisms of action being the induction of antibody-dependent cellular cytotoxicity (ADCC), primarily mediated by natural killer (NK) cells (28). Several GPC3-targeted mAbs are under development for the treatment of HCC. Among them, Codrituzumab, a humanized anti-GPC3 antibody, has been tested in clinical trials after demonstrating strong antitumor activity in preclinical HCC models (29). Therefore, we sought to determine whether the virus-encoded GPC3 is recognized by this antibody.

To assess compatibility with codrituzumab, we first evaluated the antibody’s ability to bind to virus-encoded GPC3 on the surface of infected cells. HepG2^GPC3-KO^ cells were infected with the OV at varying MOIs, followed by incubation with fluorescently labeled codrituzumab. Flow cytometric analysis showed a dose-dependent increase in codrituzumab binding to infected cells (**Figure 8A**). This binding was further validated via immunofluorescence, which demonstrated selective codrituzumab staining in OV-infected, but not mock-infected, cells (**Figure 8B**).

**Figure 8:**
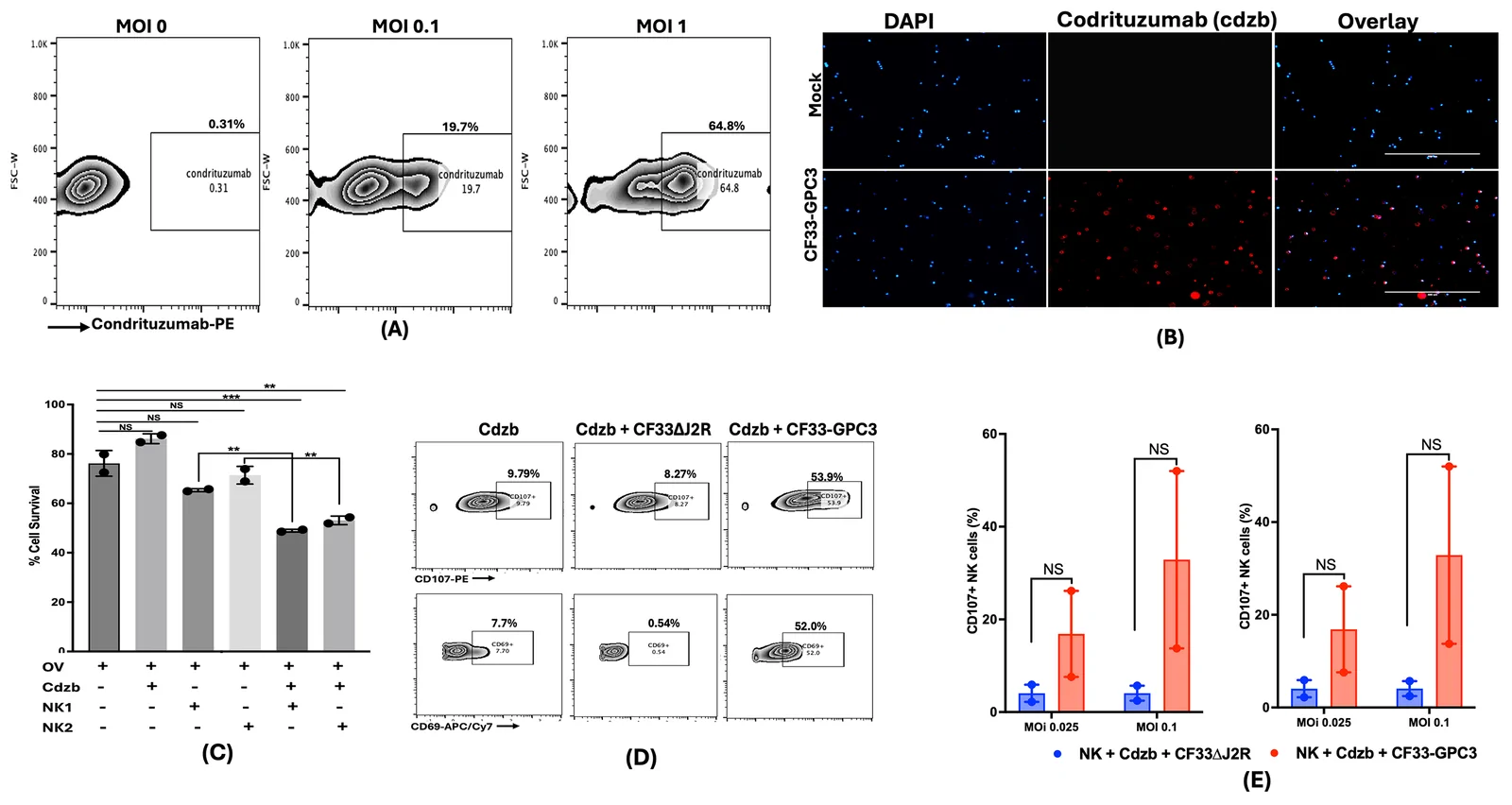
Codrituzumab (Cdzb) binds virus-encoded GPC3 and enhances NK cell–mediated cytotoxicity. **(A)** HepG2^GPC3-KO^ cells were infected with CF33-GPC3 at indicated MOIs for 18 hours, incubated with fluorescently labeled Cdzb, and analyzed by flow cytometry. **(B)** Immunofluorescence shows selective Cdzb binding to OV-infected cells. **(C)** NK cells from two healthy donors were co-cultured with OV-infected target cells in the presence or absence of Cdzb (5 µM). Cell viability was assessed 48 h later. Cdzb significantly enhanced NK cell–mediated killing of infected cells. Statistical analysis using one-way ANOVA; *p < 0.05, **p < 0.01, ***p < 0.005, ****p < 0.001, NS= not significant. **(D, E)** In a separate experiment, activation of NK cells co-cultured with target cells infected with CF33-GPC3 or control virus (CF33ΔJ2R) was assessed by surface markers (CD107, CD69). Increased activation was observed only in the presence of CF33-GPC3, which appeared to be dependent on the dose of OV. Statistical comparison in **(E)** was performed using 2way ANOVA; NS= not significant. Data in **(A, B)** are representative of 2 independent experiments and data in **(D)** are shown for one of the 2 donors. Data in **(C)** are shown using NK cells from 2 independent donors and data in **(E)** are presented as mean ± SEM for NK cells from 2 independent donors.

Next, we tested whether codrituzumab enhances NK cell–mediated killing of tumor cells displaying virus-encoded GPC3, via ADCC. Freshly isolated NK cells from two healthy donors were co-cultured with OV–infected HepG2^GPC3-KO^ cells in the presence or absence of codrituzumab. Cell viability was assessed 48 hours later. In both donors, the presence of codrituzumab significantly increased NK cell–mediated cytotoxicity compared to that in the absence of antibody (**Figure 8C**).

We further assessed NK cell activation by measuring the expression of surface activation markers: CD107 and CD69. NK cells co-cultured with CF33ΔJ2R-infected or uninfected target cells exhibited minimal activation. In contrast, co-culture with CF33-GPC3–infected targets resulted in increased expression of both activation markers. Although this trend was consistently observed in both donors, the differences did not reach statistical significance due to inter-donor variability and the limited number of donors (n = 2) (**Figures 8D, E**).

## Discussion

Glypican-3 (GPC3) is one of the most frequently overexpressed antigens in HCC while being largely absent in normal adult tissues, making it an attractive therapeutic target. Despite this, GPC3-directed strategies—including CAR T cells, bispecific T-cell engagers (BiTEs), and monoclonal antibodies—have shown only limited clinical efficacy. As with most solid tumor immunotherapies, their activity is hindered by antigen heterogeneity, low antigen density, poor T-cell infiltration, and immune escape mechanisms.

To address these barriers, we explored an OV-based strategy to deliver GPC3 directly into tumor cells, thereby increasing antigen availability for immune targeting. Our data demonstrate efficient conversion of GPC3-negative tumor cells into GPC3-positive cells following OV infection, significantly enhancing susceptibility to GPC3-directed immunotherapies. This approach builds on our prior work using an OV to deliver CD19 to solid tumors, which enabled effective targeting by CD19-directed CAR T cells and demonstrated favorable remodeling of the tumor microenvironment with enhanced T-cell infiltration (30). Similar OV-mediated antigen delivery strategies have also been reported by other groups, including mesothelin delivery in pancreatic cancer followed by CAR T cell targeting (30).

We selected GPC3 as a superior target due to its more restricted expression profile compared with CD19 or mesothelin. While CD19 targeting results in systemic B-cell depletion and mesothelin expression in mesothelial tissues raises concerns for on-target off-tumor toxicity (31, 32), GPC3 expression in adult tissues is minimal and largely confined to HCC, supporting its suitability for targeted immunotherapy.

A major limitation of GPC3-directed therapies remains its heterogeneous and often low expression within tumors. To overcome this, we engineered CF33-GPC3 to enhance both the proportion of antigen-positive cells and surface antigen density. Because wild-type GPC3 possesses oncogenic potential, enforced expression of GPC3 raises potential safety concerns. To mitigate this risk, we introduced specific mutations into GPC3 that disrupt its interaction with β-catenin, a critical mediator of its oncogenic activity, as previously reported (33). In addition, poxvirus infection induces cell-cycle arrest and ultimately results in cell death either through direct lysis or innate immune–mediated mechanisms (34). Consequently, GPC3 expression is confined to infected tumor cells that are destined for elimination, thereby minimizing potential safety risks.

GPC3-targeted CAR T cells have shown promising preclinical activity and are currently being evaluated clinically; however, their use is limited by cytokine release syndrome, neurotoxicity, and T-cell exhaustion. The AbTCR (ARTEMIS^®^) platform offers an alternative with improved safety and reduced exhaustion compared with conventional CAR T cells. Our findings suggest that OV-mediated GPC3 delivery may broaden the applicability of ECT204 by converting GPC3-low or negative tumors into GPC3-high targets, potentially extending its use beyond HCC to other solid tumors.

We further evaluated the compatibility of OV-delivered GPC3 with other therapeutic modalities, including BiTEs and the monoclonal antibody codrituzumab. *In vitro* studies confirmed that virus-encoded GPC3 is functionally recognized by both modalities, enabling T-cell activation and tumor cell killing. Given that OVs can enhance T-cell infiltration and convert immunologically “cold” tumors into inflamed “hot” tumors, we anticipate potential synergy between OV-mediated antigen delivery and BiTE therapy. However, these findings require validation in immunocompetent *in vivo* models. Similarly, our data suggest that OV-driven upregulation of GPC3 may help overcome the limited clinical efficacy previously observed with codrituzumab, potentially by increasing target density and improving immune accessibility.

A key limitation of this study is the use of NSG xenograft models, which do not fully recapitulate the complex immune microenvironment of human HCC. Although we previously demonstrated the efficacy of a related CD19-OV strategy in immunocompetent models, the present study was limited by the lack of available murine GPC3-binding reagents. Nonetheless, our *in vitro* data indicate that CF33-GPC3 induces immunogenic cell death (**Supplementary Figure 5**), suggesting potential for favorable immune modulation in an intact immune system.

In summary, our findings demonstrate that OV-mediated antigen delivery can overcome a fundamental barrier in solid tumor immunotherapy—antigen heterogeneity—by converting GPC3-negative tumors into therapeutically targetable tumors. This strategy could expand the applicability of GPC3-directed therapies, including ECT204, BiTEs, and monoclonal antibodies, and provides a strong rationale for future clinical translation in HCC and potentially other solid tumors.

## Conclusions

In conclusion, this study provides evidence that oncolytic virus–mediated antigen delivery represents a powerful strategy to overcome the critical barrier of antigen heterogeneity in hepatocellular carcinoma. By engineering CF33-GPC3, we demonstrate efficient and robust conversion of GPC3-negative tumors into GPC3-positive targets, enabling enhanced recognition and killing by multiple GPC3-directed immunotherapies, including ARTEMIS T cells (ECT204), bispecific T cell engagers, and monoclonal antibodies. The observed improvement in antitumor efficacy, both *in vitro* and *in vivo*, highlights the translational potential of this approach to expand patient eligibility and improve outcomes for GPC3-targeted therapies. Although further validation in immunocompetent models is warranted, these findings establish a strong rationale for clinical development of OV-mediated antigen delivery as a broadly applicable platform to sensitize solid tumors to targeted immunotherapies.

## Data Availability

The original contributions presented in the study are included in the article/**Supplementary Material**, further inquiries can be directed to the corresponding author/s.
