# Supplementary material for: Oncolytic virus–mediated GPC3 delivery overcomes antigen heterogeneity in hepatocellular carcinoma and enhances GPC3-directed immunotherapy
Source: Front Immunol. 2026 Sep 18;17:1894178. doi: 10.3389/fimmu.2026.1894178 (PMC13630722; doi:10.3389/fimmu.2026.1894178)
Supplement: Supplementary file 1 [file DataSheet1.pdf]

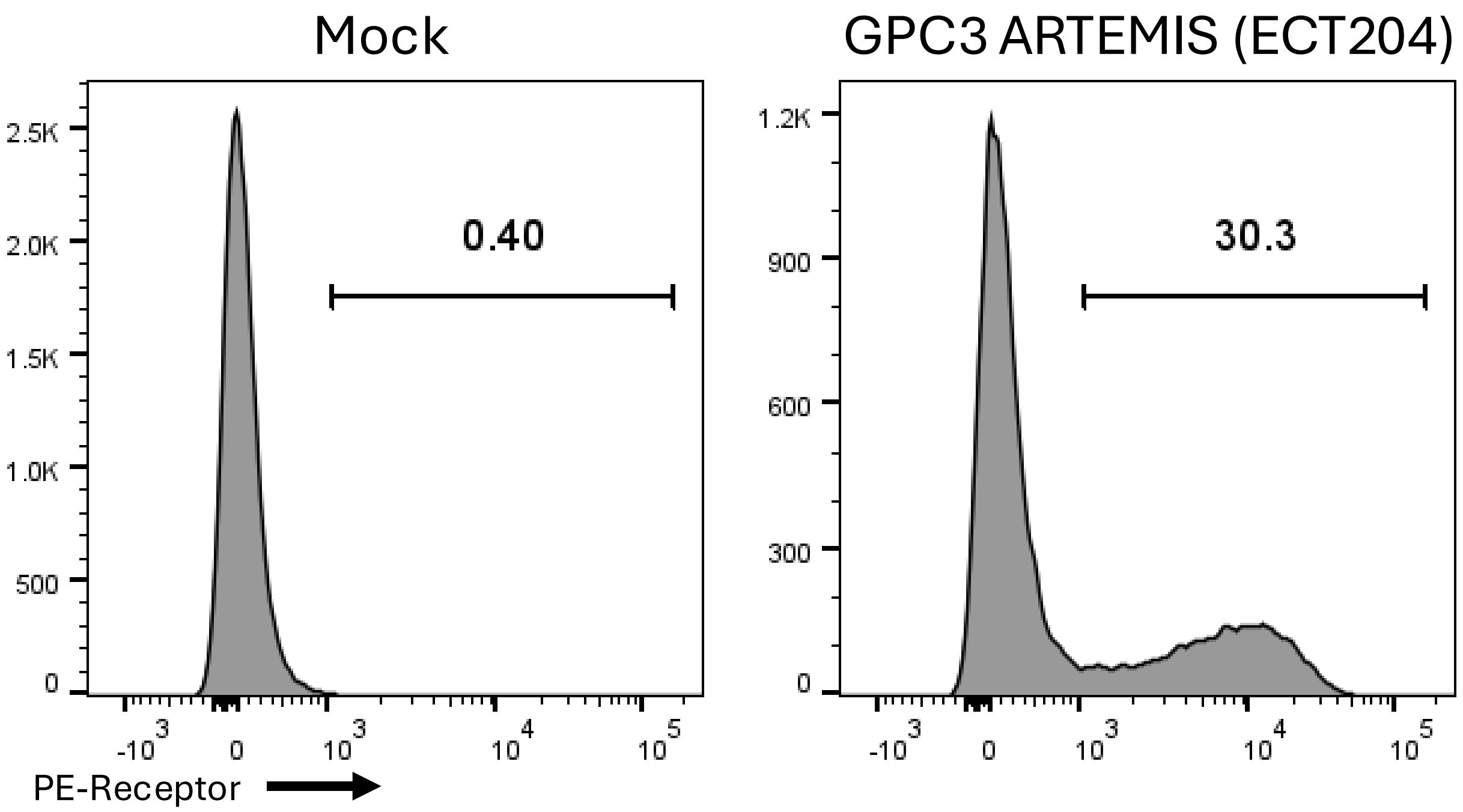

Figure S1: Confirmation of AbTCR in ECT204. Thirty per cent of the total T cells were found to be positive for the AbTCR.

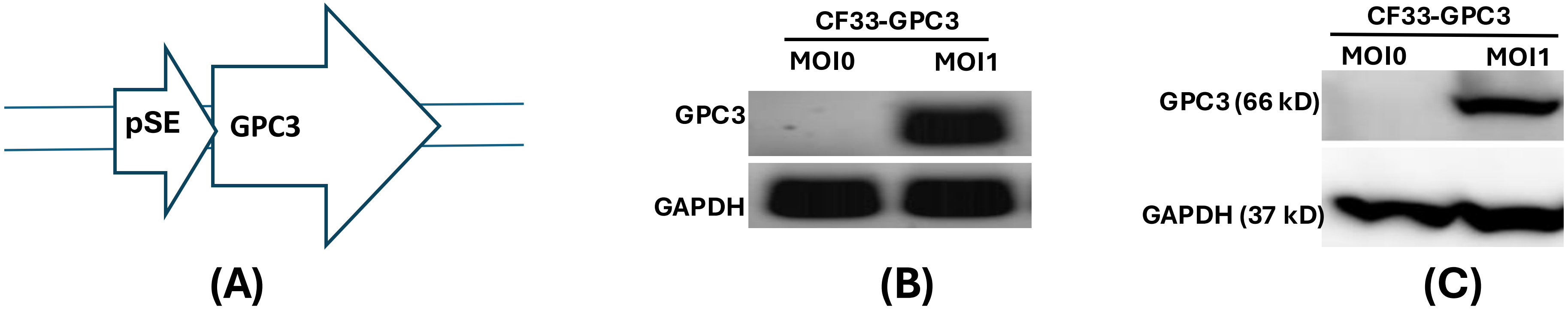

Figure S2: Confirmation of GPC3 expression from the oncolytic virus. (A) Cartoon showing GPC3 expression cassette in the OV. (B) RT pcr was perfomed on lysate from cells either mock infected (moi 0) or infected with the oncolytic virus CF33-GPC3, 24 h post-infection. The product of RT-PCR was run on an agarose gel. (B) Confirmation of GPC3 by Western blot 24 h post-infection.

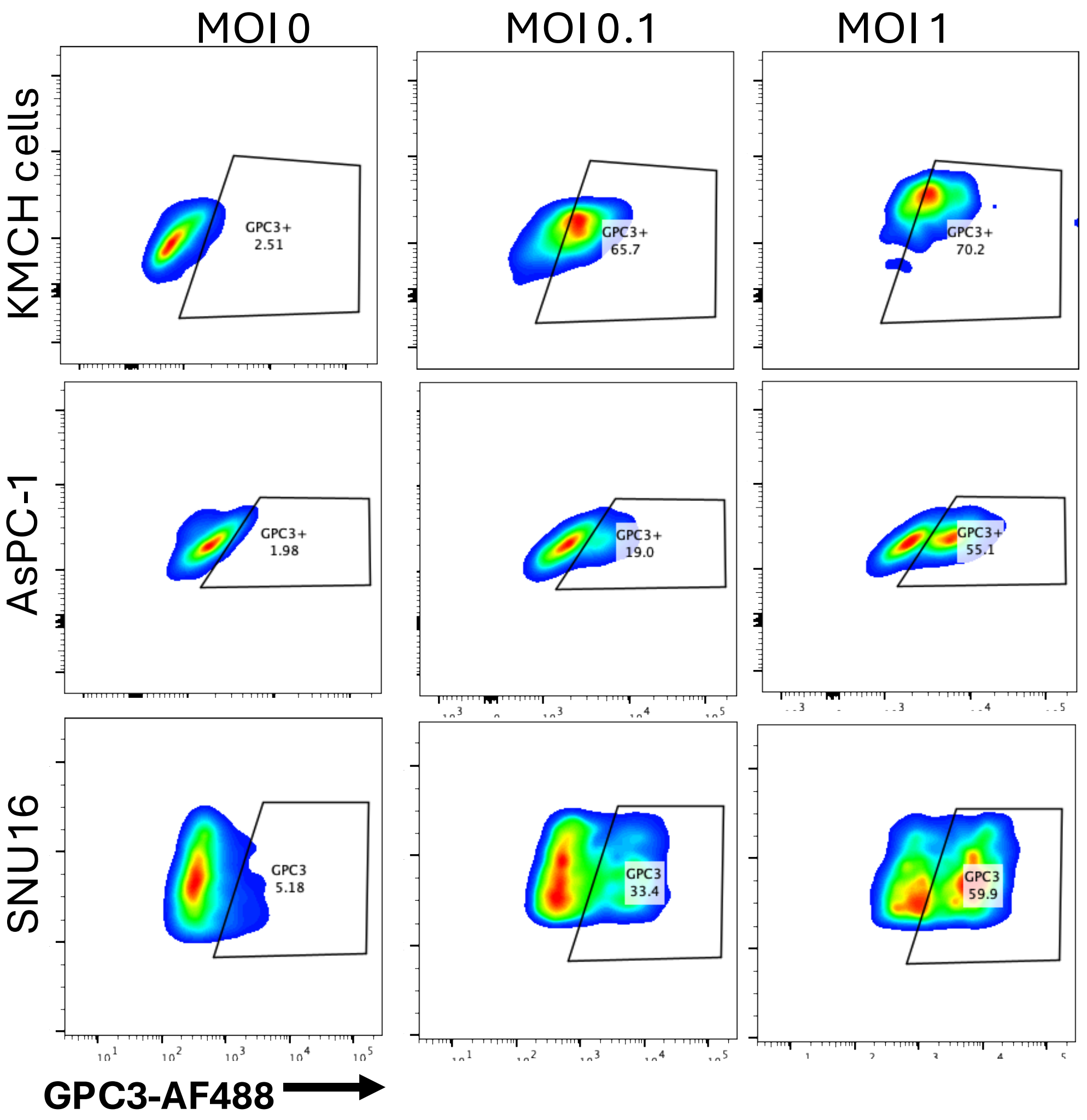

Figure S3: OV-mediated GPC3 expression in non-HCC cell lines. Cell lines representative of cholangiocarcinoma (KMCH), pancreatic cancer (AsPC1) or gastric cancer (SNU16) were infected with the OV (CF33-GPC3) at an MOI 0.1 or 1. GPC3 levels were determined by flowcytometry 18 hours post-infection.

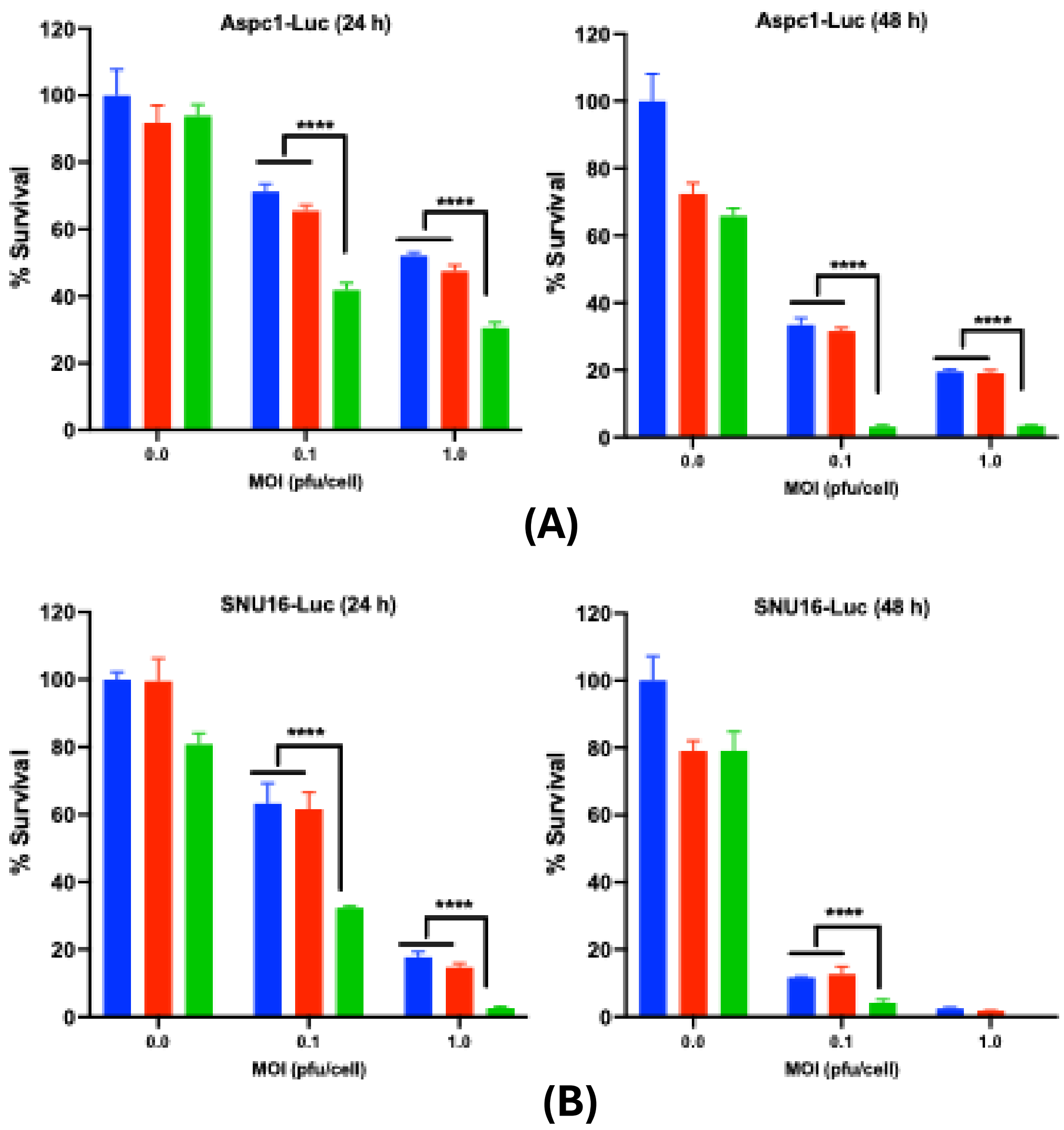

Figure S4: Cancer cells were infected with OV (CF33-GPC3) at varying MOIs and cocultured with mock or ECT204 T cells at a 1:1 ratio. Cell survival was measured 24 or 48 hours later.

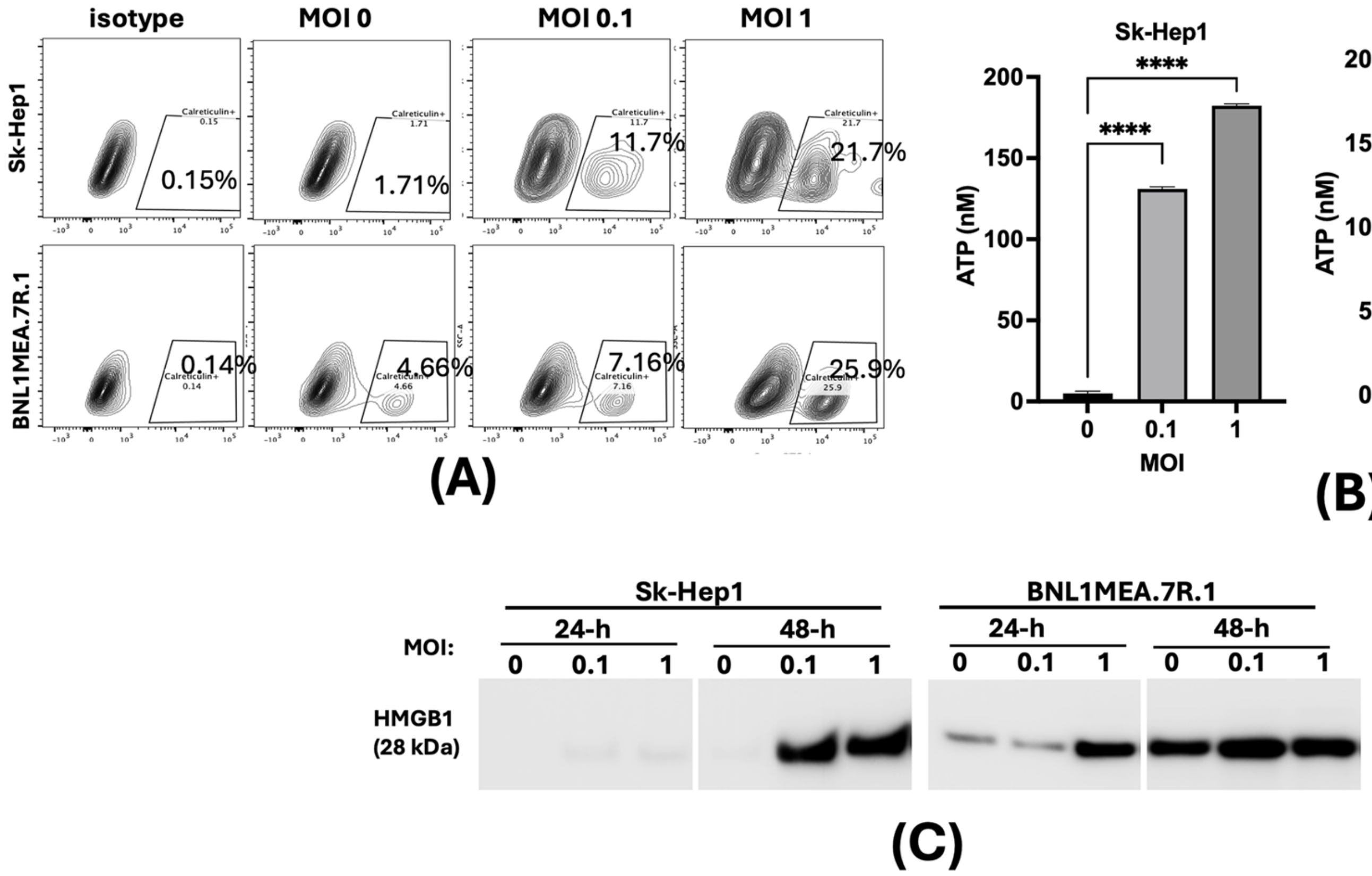

Fig. S5: The oncolytic virus CF33-GPC3 induces immunogenic cell death in HCC cells. HCC cells of human (Sk-Hep1) or murine (BNL1MEA.7R.1) origin were infected with CF33-GPC3 at indicated MOIs. (A) cells were harvested ~24 hours post-infection, stained with live/dead dye followed by staining for calreticulin. Calreticulin levels were analyzed on live cells. (B) Extracellular ATP release was quantified from culture supernatants (24 hours post-infection) using a luminescence-based ATP determination kit. Statistical comparison was performed using one-Way ANOVA; **=p<0.01, ***=p<0.005, ****=p<0.001. (C) HMGB1 release was assessed by Western blot analysis using a fixed volume (30 µL) of supernatant collected from mock- or virus-infected cells. HMGB1 was detected using a rabbit anti-HMGB1 antibody, followed by a donkey anti-rabbit secondary antibody.
